# Association between Asian Dust-Borne Air Pollutants and Daily Symptoms on Healthy Subjects: A Web-Based Pilot Study in Yonago, Japan

**DOI:** 10.1155/2016/8280423

**Published:** 2016-12-08

**Authors:** Abir Majbauddin, Kazunari Onishi, Shinji Otani, Yasunori Kurosaki, Youichi Kurozawa

**Affiliations:** ^1^International Platform for Dryland Research and Education, Tottori University, 1390 Hamasaka, Tottori 680-0001, Japan; ^2^Center for Birth Cohort Studies, Interdisciplinary Graduate School of Medicine, University of Yamanashi, 1110 Shimokato, Chuo, Yamanashi 409-3898, Japan; ^3^Arid Land Research Center, Tottori University, 1390 Hamasaka, Tottori 680-0001, Japan; ^4^Division of Health Administration and Promotion, Faculty of Medicine, Tottori University, 86 Nishi-cho, Yonago 683-8504, Japan

## Abstract

During the spring, Asian dust (AD) repeatedly makes its way to Japan, originating from drylands. We evaluated the association between AD-borne air pollutants and daily reported subjective symptoms in healthy subjects. We constructed an Internet questionnaire on daily ocular, nasal, respiratory, and skin symptoms. Forty-two healthy volunteers residents of Yonago (mean age, 33.57) were selected from the self-reporting web-based survey and recorded their symptoms between 1 and 31 of March 2013. We also collected information on levels of suspended particulate matter (SPM), particulate matter < 2.5 *µ*m (PM_2.5_), sulfur dioxide (SO_2_), and nitrogen oxide (NO_*x*_) per hour on each of those days. SPM and PM_2.5_ were the dominant pollutants recorded throughout the month. A positive correlation was observed between SPM and ocular (*r* = 0.475, *p* < 0.01), nasal (*r* = 0.614, *p* < 0.001), and skin (*r* = 0.445, *p* < 0.05) symptoms. PM_2.5_ correlations were significant for ocular (*r* = 0.428, *p* < 0.05), nasal (*r* = 0.560, *p* < 0.01), and skin (*r* = 0.437, *p* < 0.05) symptoms. Our findings provide introductory evidence of AD-borne air pollutants and their association with several bodily symptoms in healthy subjects with the implementation of a self-administrated web-based survey application.

## 1. Introduction

Asian dust (AD) is a springtime meteorological phenomenon regarded as displacement of atmospheric pollutants from Chinese and Mongolian deserts and carried by prevailing winds across Northeast Asia. These events are, however, not always accepted as a seasonal phenomenon but considered instead an environmental problem resulting from human activities, such as soil degradation, desertification, and forest reduction. In Japan, AD is frequently observed during the spring season and sometimes in late fall and is known in Japanese as “kosa” or “yellow sand.” Since the 1990s to early 2000s, AD frequency has increased gradually in East Asia, carrying with it anthropogenic metal compounds, chemicals, and spherical particles through long-range transport that passes over heavily industrialized zones in China and finally arrives in Japan [1, 2]. AD is not only problematic because of financial losses from crop damage, tree collapse, and flight cancellations due to low visibility, but also carries a major threat to human health.

Numerous comprehensive epidemiological studies have suggested that various air pollutants such as particulate matter (PM), gaseous pollutants, sulfur dioxide (SO_2_), nitrogen oxide (NO_*x*_), ozone (O_3_), volatile organic compounds, and heavy metals can exert serious effects on different organs and body systems, including the respiratory system, cardiovascular system, nervous system, urinary system, digestive system, and the developing fetus [3]. Several studies have found that an increase in particulate pollution raises the risk of daily mortality, acute myocardial infarction, and hospital visits for various other problems in different cities in Japan, Korea, and Taiwan [4–6]. In addition, some reports have shown a connection between short-term exposure to airborne particulates and acute health effects [7]. Suspended particulate matter (SPM) and particulate matter with aerodynamic diameters < 2.5 *μ*m (PM_2.5_) are complex mixtures of various solid and liquid particles and are closely related to haze among various air pollutants [8]. Studies have found that SPM can increase significantly in AD events and is strongly linked to adverse health effects in urban areas [9, 10]. Therefore, health problems triggered by air pollutants have attracted attention and have become a widespread public concern. A better understanding of the most harmful components of particulate pollution is required to design better strategies for protecting public health.

With the rapid progression of Internet technology, an appropriate use of web-based communication tools can enhance epidemiological research beyond conventional studies particularly when targeting young participants, although early web-based studies were found to be challenging because of population bias and this has been eased with the noticeable increase in the proportion of the population using the Internet [11]. Therefore, web-based questionnaires may be an attractive alternative to the traditional methods of data collection process. This present study evaluated the association between AD-borne air pollutants including SPM, PM_2.5_, SO_2_, and NO_*x*_ with daily subjective symptoms reported by healthy subjects through a self-administrated web-based survey.

## 2. Materials and Methods

### 2.1. Study Location

**Figure 1:**
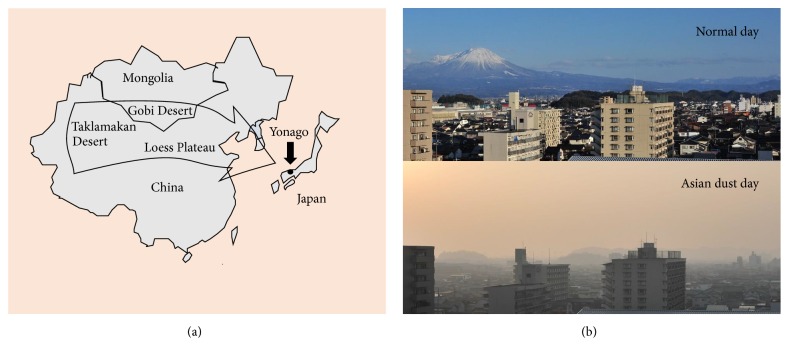
Geographical relation of the Asian dust sources (a) and the view of normal and Asian dust day (b) in Yonago, Japan.

Yonago, a medium-sized city located in the northwestern part of Tottori Prefecture, Japan, is not an industrial zone and has no major sources of air pollution, apart from one paper mill and typical motor vehicle traffic. However, Yonago often experiences AD events during the spring (Figure 1).

### 2.2. Air Pollutants and Meteorological Data

AD and meteorological information (mean temperature and relative humidity) in Yonago were obtained from the Japan Meteorological Agency. In Japan, 59 meteorological stations perform AD monitoring and determination based on visibility. Visibility is categorized into <2 km, <2 to 5 km, <5 to 10 km, and no dust. Air pollution data including levels of SPM, PM_2.5_, SO_2_, and NO_*x*_ were obtained from the Tottori Prefectural Institute of Public Health. SPM, PM_2.5_, SO_2_, and NO_*x*_ levels were recorded every hour from 1 to 31 March 2013 at two places for greater accuracy. It should be noted that SPM is defined under the National Air Quality Standard as any particle with a diameter < 10 *μ*m with a 100% cut-off; the theoretical 50% cut-off diameter for SPM is assumed to be approximately 7 *μ*m. The particle diameter of SPM measured in Japan is intermediate to those classified under PM_10_ and PM_2.5_ parameters, while daily variations of SPM are similar to those of PM_2.5_ [12, 13].

### 2.3. Web-Based Survey Application

**Figure 2:**
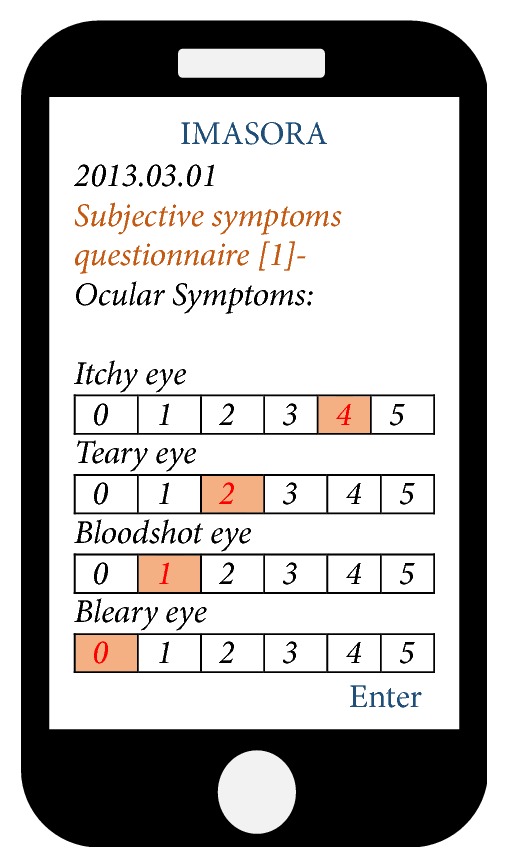
Illustration of IMASORA, a web-based health related survey application.

Prior to the collection of subjective symptom data, we constructed an Internet application (Figure 2) named “IMASORA” (images from sky observation cameras for research aerosol) for direct health related questionnaire survey of individuals on daily basis. IMASORA was made available on the Internet, App Store, and Google Play to freely download into personal computers and smartphones. Information regarding IMASORA was disseminated from 13 February 2013 via pamphlets and lectures in several public institutions in Yonago. Volunteers were requested to download the application, register with a password, and answer the questionnaire consistently every day. Key features of IMASORA were included in the design, with a list of subjective symptoms in the questionnaire, such as ocular (itchy eyes, teary eyes, bloodshot eyes, and bleary eyes), nasal (sneezing, runny nose, blocked nasal passage, and itchy nose), respiratory (sore throat, itchy throat, phlegm, and cough), and skin (itchy skin, eczema, sores, and rash). Each individual symptom was defined by a self-checking visual analogue scale, and the severity of these symptoms was measured as 0 points meaning no symptoms, 1 point meaning slight involvement, 2 points meaning mild involvement, 3 points meaning moderate involvement, 4 points meaning severe involvement, and 5 points meaning extreme involvement. The symptom score was taken as the average number of points per symptom per person. A miscellaneous questionnaire also included disease history, such as allergies, colds, influenza, and skin diseases, and drug history such as eye drops, nasal drops, skin creams or ointments, and topical creams. A daily notification was sent to the participants as a reminder during the study period. Volunteers understood the importance of this study and self-reported through mobile phones and/or personal computer.

### 2.4. Participants

A total number of 67 volunteers registered with IMASORA; among them, 42 healthy volunteers (62%), 24 men and 18 women with a mean (±SD) age of 33.57 ± 10.64 years, were selected for this study. The inclusion criteria of the subjects were restricted to those who responded every day during the study period, were nonsmokers, and had no known allergies or any other severe disease. The residential area of the participants was limited to Yonago. The exclusion criteria consisted of participants who have jobs as desk workers spending the longer part of a day indoors, potential participants undergoing any drug therapy, and those with past history of severe underlying disease. Informed consent was obtained from all individual participants included in the study. This study was approved by the ethics committee of the Faculty of Medicine, Tottori University.

### 2.5. Statistical Analysis

For statistical analysis, the levels of air pollutants and subjective symptoms scores on AD days and non-AD days were compared with Student's *t*-test. For the relationship between daily mean air pollutants and daily based subjective symptoms score was conducted by Spearman's rank-correlation with IBM SPSS software version 23 (IBM, Armonk, NY, USA). *p* < 0.05 was considered statistically significant.

## 3. Results and Discussion

**Figure 3:**
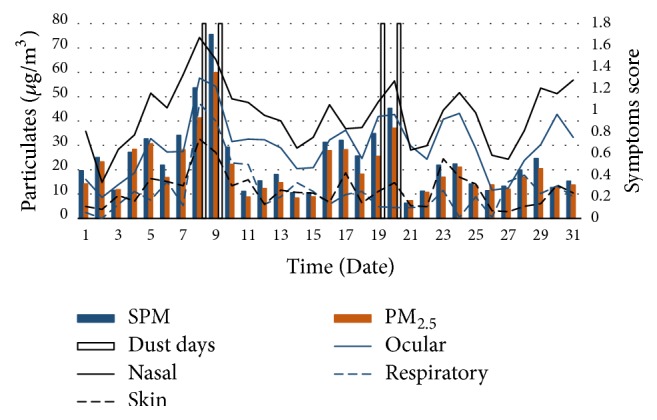
Time series of hourly mean SPM and PM_2.5_ levels with symptoms score (1–31 March 2013).

A total of 4 AD days were identified by the Japan Meteorological Agency during the study period.  Table 1 shows the air pollutants values and meteorological measurement on the AD days and non-AD days. The mean SPM and PM_2.5_ level for AD days were significantly higher (*p* < 0.001) than those for non-AD days. There was no significant difference observed between AD days and non-AD days in temperature and relative humidity. Time series of hourly mean SPM and PM_2.5_ levels with symptom scores are presented in (Figure 3). Mean levels of both SPM (±SD) (23.78 ± 14.52 *μ*g/m^3^) and PM_2.5_ (20.17 ± 11.32 *μ*g/m^3^) were recorded throughout the month. During dust days, however, SPM levels exceeded 70 *μ*g/m^3^ and PM_2.5_ levels exceeded 60 *μ*g/m^3^. In addition, the observed levels of SO_2_ (1.48 ± 1.23 *μ*g/m^3^) and NO_*x*_ (9.13 ± 4.45 *μ*g/m^3^) were relatively low. Dust and sandstorms pass over Japan throughout the year, but their levels increase from February and peak in April [14]. AD events and air pollution occurrences run concurrently, and various AD events are categorized based on their particle composition such as dust and metals from anthropogenic sources and shape (spherical, crystalline) [2]. Principally, severe AD days are usually defined based on the levels of PM_10_ and PM_2.5_, as was observed in this study, but criteria defining dust events may differ based on time and place. According to Lu et al. [15] a 10 *μ*g/m^3^ increase in both SPM and PM_2.5_ raises the risk of mortality due to cardiovascular and respiratory disease.

**Figure 4:**
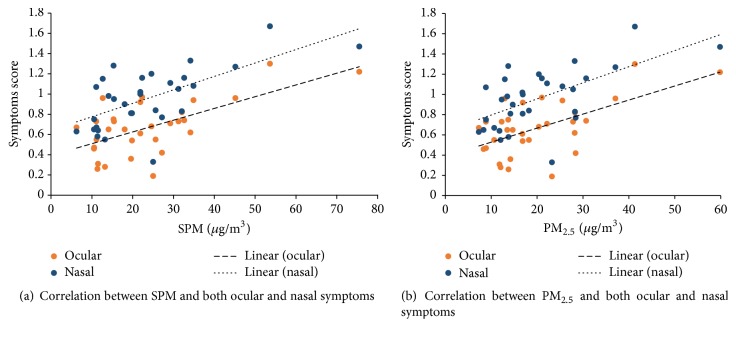
Correlation between air pollutants and subjective symptom scores.

The symptoms scores on the AD days and non-AD days are presented in Table 2. Ocular, nasal, and skin symptoms score on the AD days were significantly higher compared to that for non-AD days, except respiratory symptom.  Table 3 lists the correlation coefficient values between air pollutants and subjective symptoms. A significant correlation was observed between SPM and both nasal (*p* < 0.001) and ocular (*p* < 0.01) symptoms (Figure 4(a)). Additionally, PM_2.5_ was also found to have a significant correlation with nasal (*p* < 0.01) and ocular (*p* < 0.05) symptoms (Figure 4(b)). No significant relationship was observed between air pollutants and pharyngeal symptoms. SO_2_ and NO_*x*_ did not show any significant correlation with ocular, nasal, respiratory, or skin symptoms. However, a significant relationship was observed between skin symptoms and both SPM and PM_2.5_ (*p* < 0.05).

Our results provide introductory evidence that major air pollutants influence certain bodily symptoms in healthy subjects. A significant correlation was observed between SPM, PM_2.5_, and nasal symptoms (e.g., sneezing, runny nose, and block of nasal passages). Epidemiological studies have shown that exposure to PM is associated with a higher incidence of upper airway symptoms, such as nasal obstruction and airway irritation from particles penetrating the respiratory system [16]. The impact of AD is greater in patients with asthma, who experience lower respiratory symptoms such as cough and sputum [17]. However, we found no significant association between air pollutants and respiratory symptoms (e.g., cough, phlegm), possibly due to lack of deep penetration of PM in the lower respiratory system; notably, participants in this study were healthy, which may well have led to low scores. As a result, the effects of AD airborne pollutants could be undervalued. Our findings revealed a significant association between SPM, PM_2.5_, and ocular symptoms (e.g., itchy eyes, teary eyes). A relevant study reported that clinic visits for conjunctivitis in Taiwan increased in frequency after dust storms but did not find a significant association [18]. In contrast, Mu et al. [19] reported that tearing of the eye increased significantly in residents of desert areas in Mongolia. Seasonal confounders, such as tree pollen, should be taken into account when considering subjective symptoms reported during AD events. High levels of pollen from Japanese cedar* (Cryptomeria japonica)* have been considered a major cause of seasonal allergic complications [20]. However, studies have typically explored the effects of AD on health mainly based on PM_10_ and PM_2.5_ levels [21]. Previous studies have demonstrated significant associations between SPM and skin symptoms during dust events [9], and our findings provide similar evidence of the association between SPM, PM_2.5_, and skin symptoms.

AD particulate matter is predominantly composed of rock-forming minerals, such as quartz and feldspar, and clay minerals such as mica, kaolinite, and chlorite. Analysis of AD particles has also shown the presence of ammonium ions, sulfate ions, nitrate ions, and metallic compounds that are not considered to originate from soil erosion [14]. In our study, both SO_2_ and NO_*x*_ levels were too low to show any significant association with subjective symptoms, so we assume that the effects reported were due to physical irritation and conceivably to interactions with Japanese cedar pollinosis. To further our knowledge of the mechanisms whereby AD-borne pollutants exert effects on human health, it is crucial to characterize the interplay between the physical properties of the pollutants and the biological responses they elicit.

The paper based questionnaire has been the epidemiological mode of choice for collecting survey data up till now. Despite the fact, Ekman et al. [11] stated that the response rate is higher in web-based study compared to paper based study but the opposite has also been reported [22]. Traditional approaches to gathering information from study subjects, including face-to-face and telephone interviews and paper-and-pencil questionnaires, increasingly fail to generate qualitatively good results within the financial parameters and decreasing response rates over the last decades [23]. The implementation of web-based survey like IMASORA provides several advantages over traditional paper based or face-to-face surveys which include administration efficiency, convenience for the participant, potential cost savings, and higher data precision. IMASORA also can provide benefits of automatic validation checks incorporated with prompt alert to responders when they enter implausible or incomplete answers, reminders, and random selection of control participants. Moreover, it allows for the prediction of the health condition of general population as influenced by environmental factors on a real-time basis. From our previous experience with paper based study [9] on a similar scale we anticipated that, to conduct a large-scale epidemiological research, web-based questionnaires may provide useful insights into the answering process for volunteers and facilitate automatic collection of the so-called paradata or metadata, including date, time, and time to completion of a study. However, it has a few limitations such as response inconsistency, reluctance to participate due to Internet privacy concerns, and inconveniences (limited access to computer and Internet that may affect the willingness to participate) especially among the older people. We intend to continue this approach by strengthening IMASORA with more supplementary features to entice general population, specifically, adding several language options, repeated reminder, meteorological alert, and a simpler version that will be more convenient for all participants irrespective of age. Nevertheless, a wide range of publicizing is crucial across different areas in order to raise the number of participants. More research is necessary to better quantify the risks and benefits of conducting survey research over the Internet.

The limitations of our methodology include the small number of participants we investigated and the use of environmental and meteorological monitoring data in Yonago as a surrogate for actual exposure. In addition, we did not investigate pollen levels and organic agents such as bacteria and fungi, which may adhere to AD particles and likely influence the human body. Lastly, owing to an insufficient sample size and lack of other factors, we could not conduct multiple regression analysis to measure the relationship between air pollutants and subjective symptoms. A future study with a larger cohort, including multiple environmental factors and measurement of organic agents in Asian dust particles, may advance our understanding of the relationship between AD-borne pollutants and subjective symptoms.

## 4. Conclusion

This study measured the association between AD-borne air pollutants and several bodily symptoms in healthy subjects. Additionally, implementation of a web-based survey application may offer a feasible tool for data collection process and set the parameters for a large-scale prospective epidemiological study to address gaps in the field.

## Figures and Tables

**Table 1:** The levels of air pollutants (*µ*g/m^3^) and meteorological measurements on the Asian dust days and Non-Asian dust days.

Variable^*∗*^	Non-Asian dust days (*n* = 27)	Asian dust days (*n* = 4)	*p* value
SPM	19.56 ± 8.09	52.28 ± 17.26	<0.001
PM_2.5_	17.09 ± 6.95	40.91 ± 14.26	<0.001
SO_2_	1.35 ± 1.19	2.35 ± 1.30	0.134
NO_*x*_	9.08 ± 4.76	9.47 ± 0.96	0.873
Temperature (°C)	9.27 ± 3.13	10.03 ± 1.23	0.614
Relative humidity (%)	64.92 ± 8.53	65.25 ± 15.12	0.949

^*∗*^Values are 24-hour averages.

**Table 2:** Daily mean symptoms score on the Asian dust days and Non-Asian dust days.

Symptoms	Non-Asian dust days (*n* = 27)	Asian dust days (*n* = 4)	*p* value
Ocular	0.60 ± 0.20	1.10 ± 0.18	<0.001
Nasal	0.89 ± 0.24	1.37 ± 0.25	<0.01
Respiratory	0.12 ± 0.03	0.35 ± 0.12	0.187
Skin	0.23 ± 0.12	0.87 ± 0.25	<0.05

**Table 3:** Spearman's rank-correlation coefficient values for the association between air pollutants and subjective symptoms.

Symptoms	Ocular	Nasal	Respiratory	Skin
SPM	0.475^*∗∗*^	0.614^*∗∗∗*^	0.060	0.445^*∗*^
PM_2.5_	0.428^*∗*^	0.560^*∗∗*^	0.023	0.437^*∗*^
SO_2_	0.096	0.250	−0.016	0.297
NO_*x*_	0.118	0.163	−0.051	−0.119

^*∗∗∗*^ *p* < 0.001, ^*∗∗*^ *p* < 0.01, and ^*∗*^ *p* < 0.05.
